# Fighting Fire with Fire: Phage Potential for the Treatment of *E. coli* O157 Infection

**DOI:** 10.3390/antibiotics7040101

**Published:** 2018-11-16

**Authors:** Cristina Howard-Varona, Dean R. Vik, Natalie E. Solonenko, Yueh-Fen Li, M. Consuelo Gazitua, Lauren Chittick, Jennifer K. Samiec, Aubrey E. Jensen, Paige Anderson, Adrian Howard-Varona, Anika A. Kinkhabwala, Stephen T. Abedon, Matthew B. Sullivan

**Affiliations:** 1 Department of Microbiology, The Ohio State University, Columbus, OH 43210, USA; howard-varona.2@osu.edu (C.H.-V.); vik.1@buckeyemail.osu.edu (D.R.V.); solonenko.2@osu.edu (N.E.S.); li.918@osu.edu (Y.-F.L.); consuelogazitua@gmail.com (M.C.G.); chittick.3@osu.edu (L.C.); Jennifer.Samiec@osumc.edu (J.K.S.); aubrey.jensen9@gmail.com (A.E.J.); anderson.2805@buckeyemail.osu.edu (P.A.); ahowardv11@gmail.com (A.H.-V.); 2 EpiBiome, Inc., 29528 Union City blvd, Union City, CA 94587, USA; anikaak@gmail.com; 3 Department of Civil, Environmental and Geodetic Engineering, The Ohio State University, Columbus, OH 43210, USA

**Keywords:** Antibiotic-resistant bacteria, bacteriophage therapy, phage therapy, lysogenic conversion, prophage induction, read recruitment, shiga toxin

## Abstract

Hemolytic–uremic syndrome is a life-threating disease most often associated with Shiga toxin-producing microorganisms like *Escherichia coli* (STEC), including *E. coli* O157:H7. Shiga toxin is encoded by resident prophages present within this bacterium, and both its production and release depend on the induction of Shiga toxin-encoding prophages. Consequently, treatment of STEC infections tend to be largely supportive rather than antibacterial, in part due to concerns about exacerbating such prophage induction. Here we explore STEC O157:H7 prophage induction in vitro as it pertains to phage therapy—the application of bacteriophages as antibacterial agents to treat bacterial infections—to curtail prophage induction events, while also reducing STEC O157:H7 presence. We observed that cultures treated with strictly lytic phages, despite being lysed, produce substantially fewer Shiga toxin-encoding temperate-phage virions than untreated STEC controls. We therefore suggest that phage therapy could have utility as a prophylactic treatment of individuals suspected of having been recently exposed to STEC, especially if prophage induction and by extension Shiga toxin production is not exacerbated.

## 1. Introduction

Prophages are bacteriophage (phage) genomes that replicate alongside their bacterial host’s genome until induced to produce viral particles. This carriage state, termed a lysogenic cycle, is characteristic of temperate phages (as opposed to strictly lytic, or virulent, phages), and the prophage-carrying bacterial host is termed a lysogen. Recent reviews provide information on the diverse and impactful biology and distribution of temperate phages, along with methods for temperate phage detection [1,2,3]. One impact of temperate phage biology is lysogenic conversion: the modification of a host phenotype by prophage genes, including genes encoding bacterial virulence factors [4,5,6].

Notable among prophage-encoded virulence factors are exotoxins, such as those associated with the O157:H7 serotype of Shiga-toxigenic *Escherichia coli* (STEC) [7]. STEC O157:H7 is a polylysogenic human pathogen, often derived from ruminant gastrointestinal tracks and known for its capacity to encode two Shiga toxins, dubbed Stx1 and Stx2 [8,9]. These are generally encoded by the Shiga-toxigenic prophages 933V and 933W, respectively [10,11,12]. Of these, only the lamboid 933W prophage appears capable of inducing, and does so spontaneously [11,13,14,15,16,17]. This induction and the associated lytic cycle are a prerequisite for Shiga toxin production and release [18,19,20]. Shiga toxin release during STEC O157:H7 infection can lead to hemorrhagic colitis and hemolytic–uremic syndrome (HUS), which damages kidney nephrons of the STEC-infected human patients [20,21,22], but causes little to no pathogenesis in ruminants [23].

Certain antibiotics that induce the STEC SOS response also can induce Shiga-toxigenic prophages, resulting in new intracellular Shiga toxin production and subsequent phage lysis-associated toxin release [4,11,16,20,24,25,26]. Thus, prophage induction, in addition to bacterial lysis, drives increases of Shiga toxin within STEC-infected individuals, and prophage-inducing antibiotics therefore are not recommended for STEC treatment. Consequently, STEC killing via other non-prophage inducing methods—even lytic mechanisms, such as through infection by strictly lytic phages—should serve as viable STEC treatment. Treatment using non-Shiga-toxigenic phages (phage therapy) should not in itself give rise to an increased degree of patient exposure to Shiga toxin than would occur without such non-inductive lysis. Furthermore, lysogen killing by means that do not induce prophages should curtail future induction events, which presumably will result in less overall Shiga toxin production. 

Based on the above assumptions, we reasoned that lysis of STEC O157:H7 by strictly lytic phages might eliminate STEC O157:H7 without further contributing to Shiga toxin production. If true, then such lytic phages might be employed as a means of anti-STEC treatment, and by extension as anti-Shiga-toxigenic phage agents—in effect an anti-temperate phage form of phage therapy. 

Here we test this hypothesis through in vitro experiments designed to explore the use of strictly lytic phages, unrelated to Shiga toxin-encoding prophages, as anti-STEC bactericidal agents, in order to assess the potential impact of phage therapy on the production of Shiga-toxigenic 933W phages by *E. coli* O157:H7.

## 2. Results

### 2.1. Detecting Spontaneous Prophage Induction

From the American Type Culture Collection (ATCC—identifier ATCC43895) we acquired the STEC serotype O157:H7 whose genome sequence is published under strain EDL933 [11,12]. In order to have an up-to-date genome sequence (herein termed STEC), we re-sequenced our working strain and identified prophage regions with the online tool PHASTER [27] (Supplementary Materials). This confirmed the working strain as largely identical to the published EDL933 at 100% average nucleotide identity (ANI) with only a ~1% difference in genome length (see Supplementary Materials, Table S1). Predicted prophage content between STEC and EDL933 was also largely congruent, with the small variation observed likely due to differences in sequencing and assembly methodology (Supplementary Materials, Figure S1). 

With a fully-sequenced working strain, we then assessed spontaneous prophage induction in STEC as follows. STEC cultures were grown in triplicate for 5 h, treated with chloroform for 2.5 h to lyse the cells and release encapsidated phage DNA, and 0.2 µm-filtered to remove cells and large cellular debris. Samples were then treated with DNase to minimize free DNA and enrich for encapsidated DNA. The DNA was then extracted and sequenced, and the resulting reads were mapped to the STEC genome, including prophage regions. Given that most free bacterial DNA was removed with DNase, elevated read recruitment across the entirety of any prophage region would indicate induction and subsequent encapsidation of the prophage region(s). This read recruitment methodology is especially useful for identifying which prophages are induced within polylysogens, as previously shown [28,29,30].

**Figure 1:**
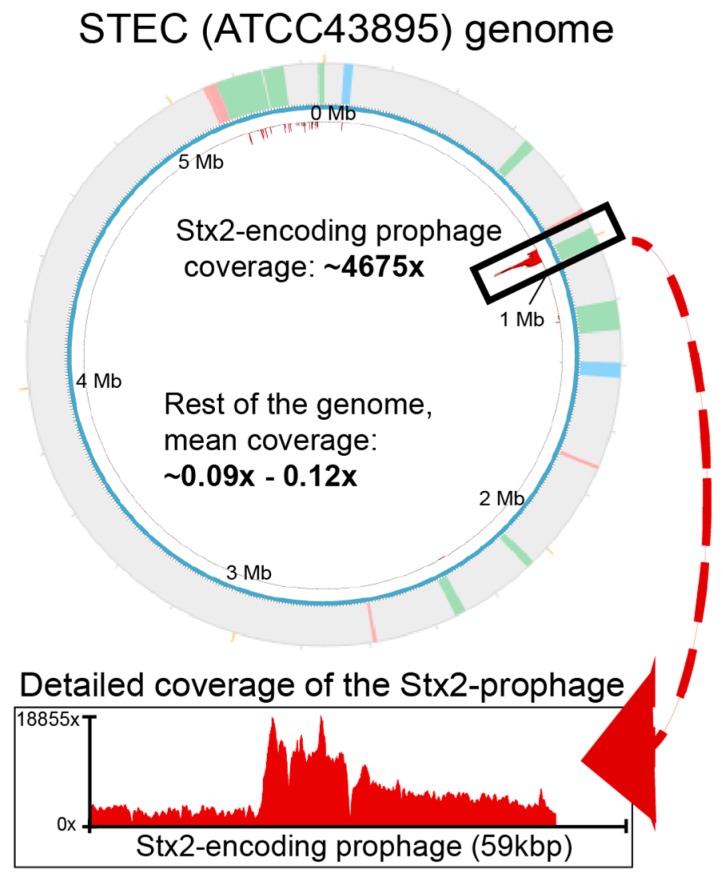
Phage 933W is the only prophage that is spontaneously induced. Shown here is the read mapping from sequenced *Escherichia coli* (STEC) cultures in biological triplicates. The circular plot represents the host genome, with the PHASTER-predicted prophages in colors (pink, red, or blue) in the outer circle, as well as the reads mapped to the entire genome. Prophage 933W is covered ~4675 times on average throughout its entire length, whereas the rest of the non-prophage and prophage genomic regions are covered, on average, 0.09 and 0.12 times, respectively. The prophage 933W region and read-mapping to such a region is amplified below the circular plot to show that the entire prophage length is covered by reads, and their proportion. Detailed information of the reads can be found in Table 1 and in the Supplementary Materials, Dataset.

Mean read recruitment coverage values were calculated per host or prophage region and normalized by the sequencing depth and the sequence length of either the 933W genomic region (59,338 bp) or the STEC genome without the 933W prophage (5,499,692 bp). This revealed that prophage 933W, which encodes the Stx2 genes and is responsible for much of STEC’s pathogenesis [20,31], had substantially higher mean coverage (4675×) than either the rest of the host genome (0.09×) or other prophage regions (0.12×), and that this elevated read coverage encompassed nearly all (95%) of the 933W genome (Figure 1, Table 1, and Supplementary Materials). We interpret this as evidence for spontaneous induction and encapsidation of 933W in this STEC strain, a finding consistent with prior work that describes prophage 933W as a highly spontaneously inducible prophage [11,13,14,15,16,17].

**Figure 2:**
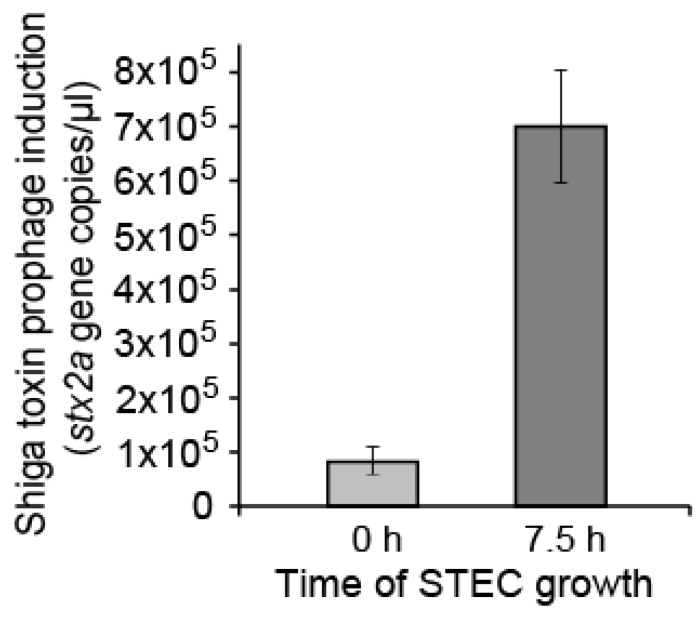
Quantification of prophage 933W induction in uninfected STEC cultures via quantitative PCR (qPCR). Primers are used against the *stx2* subunit *a* gene at 0 and 7.5 h of STEC growth. The former represents a transfer of cells from an overnight growth into fresh media, and the latter represents when cell growth is stopped and the DNA harvesting procedure begins (see Methods). The average of three biological replicates and their error is plotted on the graph. The difference between the two time points is significantly different (*t*-test, *p* < 0.05). Data from this experiment can be found in the Supplementary Materials (see Dataset).

With this qualitative screening identifying only the 933W prophage as having been induced, we sought to quantify 933W phage production as a product of spontaneous induction via a quantitative PCR (qPCR) approach targeting the Shiga toxin gene *stx2a* encoded by 933W. To this end, we grew and sampled STEC as done for the whole-genome induction screen above, and found that the prophage 933W-encoded *stx2a* copy number increased eight-fold from the start to the end of the aforementioned 7.5 h experiment (~10^5^ to 8 × 10^5^ per µL of filtrate) (Figure 2). This corroborates the sequence-based indication of prophage 933W spontaneous induction and implies ongoing induction over the course of culture incubation, since encapsidated DNA was present in somewhat smaller amounts at the start of the incubation. Prophage 933W induction, therefore, should be quantitatively reducible by preventing ongoing lysogen growth, such as may be accomplished in the course of phage therapy.

### 2.2. Fighting Prophage Induction with Phage Treatment

**Figure 3:**
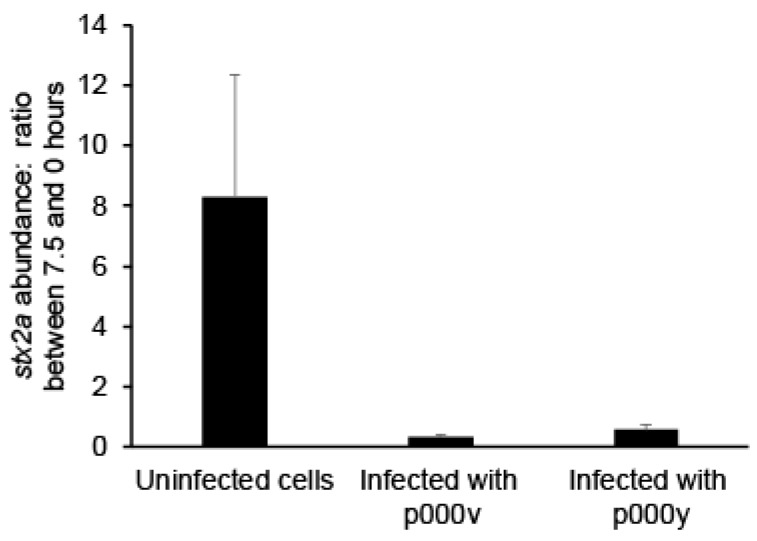
Quantification of prophage 933W induction in phage-infected STEC cultures via qPCR. The Shiga toxin (*stx2* subunit *a*) gene abundance in prophage 933W is measured at 0 and 7.5 h post-phage addition to STEC cell cultures at multiplicities of infection (MOIs) of ~4.6 (for phage p000y) and ~6.4 (for phage p000v). Represented is the ratio of such *stx2a* abundance between 7.5 and 0 h of STEC growth, using the average of the biological replicates and their error, either in the absence (left most bar in the graph) or presence (the other two bars) of phages. The differences between in the absence of phages (uninfected cells) and in the presence of phages (infected with p000v or p000y) are statistically significant (*p* < 0.05).

Given that 933W induction is known to be associated with Shiga toxin production in *E. coli* O157:H7 [20], we next considered whether treatment using exogenously supplied, strictly lytic phages could reduce lysogen numbers without exacerbating prophage induction. We used the T4-like phages p000v and p000y that we previously isolated and sequenced [32], and which we here characterized for their infection of STEC (Supplementary Materials: Figures S2 and S3, Dataset). We then grew and sampled STEC as described above, except we also added either of these exogenous phages to the STEC culture at ratios of roughly 4–6 phages per target bacterium (multiplicity of infection (MOI): ~4–6), where initial infective titers were ~6.4 × 10^8^ and ~4.6 × 10^8^ plaque-forming units per ml for phages p000v and p000y, respectively. Indeed, by the end of the experiment, addition of these phages had decreased the levels of 933W prophage induction, as quantified by qPCR. Namely, while the qPCR-measured ratio of *stx2a* copies per µL between 7.5 and 0 h was ~8 without phage (Figure 2), with the addition of phages p000v and p000y it decreased to ~0.3× and ~0.4×, respectively (Figure 3, Table 2). Thus, these results show that exogenous, strictly lytic phages reduce *stx2a* copies (a proxy for 933W prophage induction) and suggest that Shiga toxin production would also be reduced, due to both no further stimulation of prophage induction upon lytic phage infection, on the one hand, and reduction in the number of lysogens present on the other.

## 3. Discussion

The primary question regarding the potential for using phage therapy to treat pathogenic lysogens is whether such treatment might exacerbate patient exposure to toxins produced upon prophage induction. For Shiga-toxigenic *E. coli* O157:H7 in particular, Shiga toxin production and release is associated with prophage induction, mostly prophage 933W [14,19,33,34,35]. Consequentially, treatment options for STEC infections are largely supportive rather than antimicrobial for tackling Shiga toxin production and patient exposure [36,37]. 

**Figure 4:**
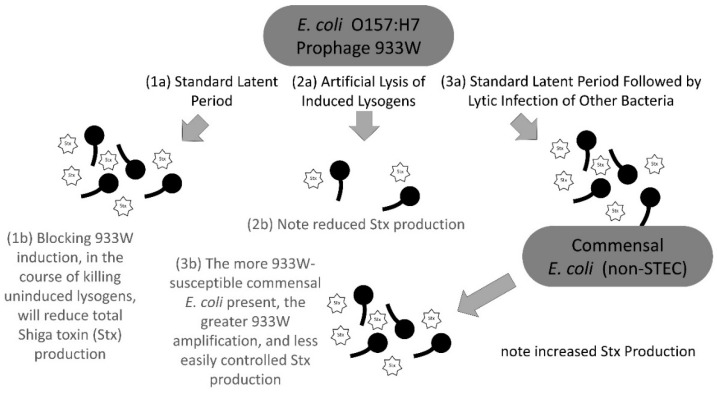
Different routes towards Shiga toxin (Stx) release: (1) 933W prophage induction followed by normal lytic cycles; (2) artificial lysis, for example by exogenous phage, of induced lysogens, resulting in truncated lytic cycles; and (3) subsequent lytic infection of non-Shiga toxigenic *E. coli* strains giving rise to more lytic cycles. Greater Stx production (stars in the figure) can occur given artificial induction of *E. coli* O157:H7 lysogens, but this both is not explicitly illustrated in the figure and is distinct from artificial lysis of already induced lysogens (2). Given the linkage between 933W induction and Shiga toxin production, the killing of *E. coli* O157:H7 lysogens without inducing the 933W prophage should result in reductions in future 933W induction events (Figure 3 and Table 2) along with subsequent reductions in Shiga toxin production.

There are three related routes by which Shiga toxin exposure could occur (Figure 4). First, the standard route (point 1a, Figure 4) is through prophage induction, resulting in Shiga toxin (Stx) gene expression followed by Shiga toxin release via phage-induced bacterial lysis [20,38]. Thus, it is crucial to avoid treatments that can lead to additional prophage induction, which can result from certain antibiotic uses [20,39]. 

A second route of Shiga toxin release (point 2a, Figure 4) may occur via artificial lysis of induced lysogens by exogenous phages, if such lysogens are capable of becoming infected and sustaining a second bacteriolytic phage infection. This could accelerate cell lysis and thus toxin release if the exogenous phage has a faster replication cycle or is otherwise competitively superior to the prophage. Alternatively, co-infection by an exogenous phage and induced prophage may confound either of the phages’ replication cycle, thereby delaying the time to cell lysis. Both instances could reasonably attenuate toxin production overall due to the reduction of either the duration or the efficiency of prophage expression, thus reducing toxin translation. 

A third route (points 3a and 3b, Figure 4) may occur when the induced and then released Shiga-toxigenic temperate phage lytically infects other *E. coli* not already lysogenized by Shiga-toxigenic phages, which would consequently enable these non-STEC bacteria to express Shiga toxin [40,41,42]. This latter route may not be easily blocked if sufficient numbers of these alternative hosts are present and support substantial Shiga-toxigenic phage population growth (point 3b, Figure 4). Based on our results, Shiga-toxin amplification from such infections may, however, be curtailed by intervening with phage treatment prior to lysogen induction and resulting Shiga-toxigenic phage production.

Here we have confirmed that an exogenously supplied obligately lytic phage “treatment” can interfere with the spontaneous production of Shiga-toxigenic prophages encoded by an *E. coli* O157:H7 strain. The mechanism of reduction in prophage induction presumably is due to the killing of prophage-containing lysogens, apparently prior to natural or artificially triggered induction. It remains unconfirmed, however, how such phage treatment will impact Shiga toxin production or release. It is likely, though, as Shiga-toxogenic prophage induction is tightly coupled to Shiga toxin production [20,38], that phage treatment of *E. coli* O157:H7-exposed patients at the very least should mitigate Shiga toxin production by killing prophage-carrying Shiga-toxigenic lysogens.

To most effectively treat such Shiga-toxigenic pathogens, future research will need to explore several areas. First, it is not known to what extent, or with what variability, different treatment phages can impact the lytic cycles of already-induced lysogens (Figure 4, 2a). Additionally, recent research with environmental phage–host systems depicts the importance of also considering the host’s response, given that they are often the ones driving the infection outcomes instead of the phages [43,44,45]. Second, it needs to be determined whether rapid treatment-phage-mediated *E. coli* O157:H7 killing is achievable in situ. It is likely, however, that achieving relatively high in-situ phage titers, e.g., 10^8^ per ml or higher [46], would be required to attain such rapid treatment-phage impact, while substantial reductions in overall Stx production will require early initiation of treatment, such as in response to suspected rather than confirmed pathogen exposure (i.e., so-called “inundative” and prophylactic phage treatment, respectively). Third, while phage therapy is generally considered as a safe treatment, given the relative lack of toxicities and side effects, especially during oral delivery [47], further verification is needed prior to generalizing clinical implementation. Generally, these issues point to a broader “pharmacologically aware” approach to the development of any phage-based *E. coli* O157:H7 infection treatment, involving iteration between continued in vitro and in vivo as well as in silico studies. In this vein, the observations reported here are consistent with *E. coli* O157:H7 phage treatment likely not giving rise to negative outcomes, as can stem from the exacerbation of 933W prophage induction. 

## 4. Materials and Methods

Raw data is provided in the Dataset, and additional methods can be found in the Supplementary Materials. 

### 4.1. Bacterial Strain and Phages Used in This Study

The Shiga-toxigenic *E. coli* serotype O157:H7 (STEC) used in this study was obtained from the American Type Culture Collection (ATCC) under identifier 43895, which is published as EDL933 under GenBank accession numbers CP008957 and CP008958 [11,12]. The T4-like Myoviridae phages p000v and p000y are described elsewhere [32], and can be found in the Cyverse data repository [48] under DOI 10.7946/P2HP89 (https://www.doi.org/10.7946/P2HP89), and in GenBank under accession numbers MK047717 and MK047718, respectively.

### 4.2. Cell Growth

Bacteria were streaked onto TSA (Tryptic Soy Agar, 40 g/L, Ward’s Cat. 38-1010) plates from glycerol stocks, grown overnight at room temperature (RT), and held at 4 °C. Single colonies were then inoculated in TSB (Tryptic Soy Broth, 30 g/L, Ward’s Cat. 38-1012) and grown shaking at ~200 rpm at 37 °C overnight.

### 4.3. Lysates for Phage Amplification

An overnight bacterial culture was diluted 1:50 in fresh TSB and grown shaking at ~200 rpm at 37 °C until the optical density (OD) reached ~0.3 (2.94 × 10^8^ CFUs/mL). Phages were added to 10–50 mL of the host culture at a low MOI (10^-6^–0.1), and incubated shaking at ~200 rpm at 37 °C for ~5 h. Chloroform was added to the infection at 1% (*v/v*) and incubated shaking at ~70 rpm at RT for 2 h. The chloroform was allowed to settle for 30 min, and the aqueous phase was 0.2 μm-filtered to remove any remaining cells. Some lysates were also concentrated via polyethylene glycol (PEG)-precipitation. For those, both NaCl (6.5 g) and PEG-8000 (10 g) were added per 100 mL lysate. This was incubated overnight at 4 °C, then centrifuged at 10,000 *g* in a Beckman J2-MC centrifuge (Beckman Coulter, Brea, CA, USA) for 10 min. The supernatant was removed, and the pellet resuspended in phage buffer (4 g NaCl, 0.1 g gelatin, 10 mL 1 M Tris Base (pH 7.6), and 1 mL 1 M MgSO_4_ per L).

### 4.4. DNA Extraction

The STEC strain obtained from ATCC (ATCC43895) was sequenced. For that, its genomic DNA was extracted using the ZymoBIOMICS DNA mini kit (Zymo Research, Irvine, CA, USA) following the manufacturer’s protocol. Similarly, sequenced phage infections and phage-free cultures from which prophage induction was assessed were also sequenced. The DNA of these samples was extracted using the Phage DNA Isolation Kit (Norgen Biotek Corp., Thorold, ON, Canada) following the manufacturer’s protocol. Any remaining host DNA was degraded by adding 10 µL (20 U) of DNase I from the RNase-free DNase I kit (Norgen Biotek Corp., Thorold, ON, Canada) prior to proteinase K treatment. Bacterial host DNA concentrations were quantified using the Qubit 3.0 Fluorometer and the Qubit dsDNA High Sensitivity Kit (Thermo Fisher Scientific, Waltham, MA, USA). 

### 4.5. Library Preparation and Illumina Miseq Sequencing of Phage and Host Genomes

Extracts from the previous step were prepared for sequencing on the Illumina MiSeq platform (Illumina, San Diego, CA, USA) using the Nextera XT Library Preparation Kit (Illumina, San Diego, CA, USA) according to the manufacturer’s protocol (Part # 15031942, revision D). The magnetic bead normalization step was replaced with a manual normalization step, based on library concentration and average size as measured by the Qubit 3.0 Fluorometer and Qubit dsDNA High Sensitivity Kit (Thermo Fisher Scientific, Waltham, MA, USA) and the Fragment Analyzer (AATI, Ankeny, IA, USA), respectively. Paired-end sequencing was performed using the MiSeq Reagent v3 (600 cycle) kit (Illumina, San Diego, CA, USA). 

### 4.6. Whole-Genome Sequencing and Read-Mapping to Assess Prophage Induction

Two sample types were obtained for sequencing whole bacterial and phage genomes, mapping reads to such genomes, and assessing prophage induction: phage-free and phage-infected bacterial cultures. The phage-infected samples were lysates grown as described in the “Lysates for Phage Amplification” section. The phage-free samples were mock lysates prepared as the infection samples, but without phages and in 30 mL containing 3.5 mL of phage buffer. After DNA extraction and library preparation procedures as described, samples were sequenced via the MiSeq technology described.

### 4.7. Read Mapping and Visualization of Prophage Induction

Reads from each of the lysates were mapped to the STEC and respective phage genome using the Burrows–Wheeler Aligner (BWA) [49] version 0.7.13, with default parameters. The resulting SAM files were converted to BAM files using samtools [50] version 1.3.1. Coverage across either phage or host genome was calculated using the Bayesian Analysis of Macroevolutionary Mixtures (BaMM) [51], software version 1.4.1, with the parse tool and the “tpmean” setting. Coverage values were then normalized by the number of reads that mapped to the virulent phages or STEC (i.e., the sequencing depth), as inferred by the samtools version 1.3.1 flagstat tool, and by the genome length of either prophage 933W, the virulent phages, or STEC without the prophage 933W. All depth- and length-normalized coverage values were then multiplied by 10^11^ to derive more comprehensible whole-genome coverage values. Coverage values per base were visualized by creating bedgraph files, using the bedtools [52] version 2.27.1 package and the genomecov -bg option. These bedgraph files were then uploaded to the Integrative Genomics Viewer (IGV) version 2.4.6 package [53] and Circos [54] version 0.69.

### 4.8. qPCR of Phage Lysates’ DNA

The OD of an overnight bacterial culture was read to determine the volume containing 10^10^ cells, which was then added to 100 mL of TSB. This was grown shaking at ~200 rpm at 37 °C; the OD was read after ~30 min and then every 10 min until the reading was 0.25–0.3. Phages were added to 2–5 mL of the host culture at MOIs lower than 0.1 or close to 6. A 0.3–1 mL sample was taken immediately and 0.2 μm-filtered to remove any cells, then stored at 4 °C. The infected culture was then incubated shaking at ~200 rpm at 37 °C for 5 h. Chloroform was added to the infection at 1% (*v/v*) and incubated shaking at ~70 rpm at RT for 2.5 h. The chloroform was allowed to settle for 20 min, and the aqueous phase was 0.2 μm-filtered to remove cells. Another 0.3–1 mL sample was taken and stored at 4 °C. DNA was extracted from the two filtered samples. First, the viral DNA was inactivated using DNase in a ratio of 1 µL of DNase to 9 µL of sample. Ethylenediamine tetraacetic acid (EDTA) and Ethylene glycol tetraacetic acid (EGTA) were added at 100 mM to inactivate the DNase. After the DNA was inactivated, extraction was continued using a Wizard DNA Clean-up Kit (Cat. #A7181, Promega Corporation, Madison, WI, USA). Then, 1 mL of DNA clean-up resin was added to each sample, and they were mixed by inversion. The samples were put into a syringe and pushed through a Wizzard minicolumn (Cat. #A7211, Promega Corporation, Madison, WI, USA), followed by the addition of 2 mL 80% isopropanol pushed through the column, 1 mL at a time. Samples were centrifuged for 2 min at 10,000 *g* to remove any excess isopropanol. Each sample was eluted in 50 µL of Tris EDTA (TE) that had been warmed to 80 °C. At the addition of TE, each sample was briefly vortexed and then centrifuged at 10,000 *g* for 30 s to elute the DNA. The samples were then analyzed for their prophage content via qPCR.

To run the qPCR, 2 µL of DNA extracted from a phage lysate was used as the template in the 15 µL qPCR reaction that contained 1× Perfecta SYBR Green FastMix (Quanta Biosciences, Gaithersburg, MD, USA) and 300 nM of each of the forward and reverse primers targeting prophage 933W (gene *stx2a* forward primer: 5′-ATGTGGCCGGGTTCGTTAAT-3′; reverse primer: 5′-TGCTGTCCGTTGTCATGGAA-3′). The qPCR reaction was carried out with the following thermocycler conditions: initial denaturation and enzyme activation at 95 °C for 5 min, 40 cycles of denaturation at 95 °C for 30 s, annealing at 57 °C for 30 s, and extension at 72 °C for 30 s, followed by one cycle of 95 °C for 15 s, 57 °C for 15 s, and 95 °C for 15 s for the dissociation curve. Fluorescence signal was collected at the end of the extension step and of the ramping period of dissociation curve. Serial dilution of the genomic DNA of strain STEC was used to generate the standard curve (R^2^ > 0.99). 

## 5. Conclusions

With the rise in antibiotic resistance in bacterial pathogens, phage therapy presents a promising alternative for treatment. Importantly, though, many of pathogens contain prophages that not only are commonly the source of pathogenesis [3,6], but have also been shown to impact cell-virus communication systems [55]. Thus, as phage therapy advances, it will be important to investigate the impacts of exogenous phages on prophage induction. Here we have provided a first step towards such investigation with whole-genome sequencing and PCR-based quantification approaches that enable a genome-wide view and quantification of what prophages are induced under both exogenous phage-free and phage-rich environments. Future work should investigate the levels of the Shiga toxin under such scenarios, as well as the impacts of phage treatment plus stressors that can induce lysogens, such as antibiotics. Additionally, research from environmental phage-host systems provides invaluable insight into ‘phage-host biology’ in nature by showing that even in prophage-free bacteria exogenous phages are not always efficient at infecting [44,45]; important when considering phage candidates for therapy. Altogether, advancing knowledge of phage-prophage-host interactions should provide a baseline for engineering phages [56,57,58] as well as inform what phages to choose to make phage cocktails that can eradicate bacterial pathogens.

## Figures and Tables

**Table 1:** Coverage of uninfected ATCC43895 (STEC)’s prophage and non-prophage regions. Represented is the raw coverage, the normalized coverage (to sequencing depth and region length), and the final transformed coverage (multiplied by 10^11^ for better reading) of each of the 17 prophages and the non-prophage regions of STEC, in biological triplicates.

Lysate	Prophage or Not?	Genomic Entity	Raw Coverage	Coverage Normalized by Sequencing Depth and Entity Length	Final Adjusted Coverage (Raised to 10^11^)
No phage control ATCC43895 (STEC)-Replicate 1	Prophages	#1-58370-85143	0.069	5.69 × 10^−14^	0.01
		#2-648527-680910	0.067	5.50 × 10^−14^	0.01
		#3 and 4-911029-938407 bp	0.835	6.89 × 10^−13^	0.07
		#5 (Stx2)-973564-1032902 bp	585.776	4.41 × 10^−8^	4407.33
		#6 and 7-1202175-1293616 bp	2.606	2.15 × 10^−12^	0.22
		#8-1390536-1436457 bp	0.018	1.47 × 10^−14^	0
		#9-1708731-1719671 bp	0.002	1.57 × 10^−15^	0
		#10-2054278-2078426 bp	0.167	1.38 × 10^−13^	0.01
		#11 (Stx1)-2302225-2335340 bp	8.057	6.65 × 10^−12^	0.66
		#12-2579647-2589259 bp	0	0	0
		#13, 14 and 15-5103469-5282316 bp	1.229	1.01 × 10^−12^	0.1
		#16-5286283-5348617 bp	0.274	2.26 × 10^−13^	0.02
		#17-5449904-5468395 bp	0.24	1.98 × 10^−13^	0.02
	Non-prophage	Host genome, non-prophage	0.852	7.04 × 10^−13^	0.07
No phage control ATCC43895 (STEC)-Replicate 2	Prophages	#1-58370-85143	0.15	8.91 × 10^−14^	0.01
		#2-648527-680910	0.514	3.05 × 10^−13^	0.03
		#3 and 4-911029-938407 bp	1.883	1.12 × 10^−12^	0.11
		#5 (Stx2)-973564-1032902 bp	881.779	4.77 × 10^−8^	4774.17
		#6 and 7-1202175-1293616 bp	4.605	2.74 × 10^−12^	0.27
		#8-1390536-1436457 bp	0.118	7.00 × 10^−14^	0.01
		#9-1708731-1719671 bp	0.22	1.30 × 10^−13^	0.01
		#10-2054278-2078426 bp	0.372	2.21 × 10^−13^	0.02
		#11 (Stx1)-2302225-2335340 bp	14.645	8.70 × 10^−12^	0.87
		#12-2579647-2589259 bp	0.097	5.73 × 10^−14^	0.01
		#13, 14 and 15-5103469-5282316 bp	2.283	1.36 × 10^−12^	0.14
		#16-5286283-5348617 bp	0.437	2.59 × 10^−13^	0.03
		#17-5449904-5468395 bp	0.562	3.34 × 10^−13^	0.03
	Non-prophage	Host genome, non-prophage	1.743	1.04 × 10^−12^	0.1
No phage control ATCC43895 (STEC)-Replicate 3	Prophages	#1-58370-85143	0.226	1.48 × 10^−13^	0.01
		#2-648527-680910	0.299	1.96 × 10^−13^	0.02
		#3 and 4-911029-938407 bp	1.379	9.03 × 10^−13^	0.09
		#5 (Stx2)-973564-1032902 bp	811.189	4.84 × 10^−8^	4844.55
		#6 and 7-1202175-1293616 bp	4.466	2.93 × 10^−12^	0.29
		#8-1390536-1436457 bp	0.089	5.82 × 10^−14^	0.01
		#9-1708731-1719671 bp	0.117	7.66 × 10^−14^	0.01
		#10-2054278-2078426 bp	0.399	2.61 × 10^−13^	0.03
		#11 (Stx1)-2302225-2335340 bp	13.974	9.15 × 10^−12^	0.92
		#12-2579647-2589259 bp	0.277	1.82 × 10^−13^	0.02
		#13, 14 and 15-5103469-5282316 bp	2.031	1.33 × 10^−12^	0.13
		#16-5286283-5348617 bp	0.345	2.26 × 10^−13^	0.02
		#17-5449904-5468395 bp	0.541	3.54 × 10^−13^	0.04
	Non-prophage	Host genome, non-prophage	1.681	1.10×10^−12^	0.11

**Table 2:** Summary of the prophage induction quantification obtained by qPCR in uninfected and infected STEC cultures, as presented in Figure 2 and Figure 3.

*Stx2a* Copies Per µL During STEC Growth with and without Phage				
Phage	0 h	7.5 h	Ratio	MOI
None	8.39 × 10^4^	6.97 × 10^5^	8.31	NA
p000v	6.93 × 10^3^	2.09 × 10^3^	0.30	6.43
p000y	8.00 × 10^3^	2.88 × 10^3^	0.36	4.61

## Supplementary Materials

The following are available online at http://www.mdpi.com/2079-6382/7/4/101/s1, Figure S1: Prophages in STEC (ATCC43895) and EDL933, and comparison between them; Figure S2: Liquid-based infection of STEC by phages p000v and p000y; Figure S3: Adsorption of p000v and p000y to ATCC43895 (STEC); Table S1: Comparison between ATCC43895 (STEC) and EDL933.
